# Learning difficulties in school children: health and education professionals’ perceptions

**DOI:** 10.1590/0034-7167-2023-0074

**Published:** 2024-04-22

**Authors:** Pamela Camila Fernandes Rumor, Michelle Kuntz Durand, Jeane Barros de Souza, Janaina Medeiros de Souza, Adriana Bitencourt Magagnin, Ivonete Teresinha Schülter Buss Heidemann

**Affiliations:** I Universidade Federal de Santa Catarina. Florianópolis, Santa Catarina, Brazil; II Universidade Federal da Fronteira Sul. Chapecó, Santa Catarina, Brazil

**Keywords:** Child Health, School Health Services, Low School Performance, Learning Disabilities, Primary Health Care, Salud Infantil, Servicios de Salud Escolar, Rendimiento Escolar Bajo, Discapacidades Para el Aprendizaje, Atención Primaria de Salud, Saúde da Criança, Serviços de Saúde Escolar, Baixo Rendimento Escolar, Deficiências da Aprendizagem, Atenção Primária à Saúde

## Abstract

**Objectives:**

to understand health and education professionals’ perceptions regarding children’s learning difficulties in public schools.

**Methods:**

qualitative research, of the participatory action type, linked to Paulo Freire’s Research Itinerary. Forty-five professionals participated, through interviews and a Virtual Culture Circle. The analysis was developed through careful reading, reflection and interpretation of highlighted topics.

**Results:**

professionals discussed the (in)visibility of learning difficulties, strategies and resources in the educational sector and the search for solutions in the health sector. It was found that the production of complaints related to school learning is attributed predominantly as an individual problem of children or their family, exempting the educational institution from this process.

**Final Considerations:**

greater investment in professional training and development policies is urgently needed to facilitate coordination between sectors, with a view to overcoming outdated pedagogical and health models.

## RESUMO

**Objetivos:**

conhecer as percepções dos profissionais da saúde e da educação acerca da dificuldade de aprendizagem de crianças da rede pública de ensino.

**Métodos:**

pesquisa qualitativa, do tipo ação participante, articulada ao Itinerário de Pesquisa de Paulo Freire. Participaram 45 profissionais, por meio de entrevistas e um Círculo de Cultura Virtual. A análise desenvolveu-se na leitura cuidadosa, reflexão e interpretação dos temas destacados.

**Resultados:**

os profissionais dialogaram sobre a (in)visibilidade da dificuldade de aprendizagem, as estratégias e os recursos do setor educacional e a busca por resolutividade no setor saúde. Verificou-se que a produção das queixas relacionadas à aprendizagem escolar está atribuída predominantemente como problema individual da criança ou de sua família, isentando a instituição educacional desse processo.

**Considerações Finais:**

é premente maior investimento em políticas de formação e aperfeiçoamento dos profissionais para propiciar a articulação entre os setores, com vistas a superar modelos pedagógicos e de saúde ultrapassados.

## RESUMEN

**Objetivos:**

comprender las percepciones de los profesionales de la salud y la educación sobre las dificultades de aprendizaje de los niños en las escuelas públicas.

**Métodos:**

investigación cualitativa, del tipo acción participativa, vinculada al Itinerario de Investigación de Paulo Freire. Participaron 45 profesionales, a través de entrevistas y un Círculo Virtual de Cultura. El análisis se desarrolló a través de una atenta lectura, reflexión e interpretación de los temas destacados.

**Resultados:**

los profesionales discutieron la (in)visibilidad de las dificultades de aprendizaje, las estrategias y los recursos en el sector educativo y la búsqueda de soluciones en el sector salud. Se encontró que la producción de quejas relacionadas con el aprendizaje escolar se atribuye predominantemente como un problema individual del niño o de su familia, eximiendo a la institución educativa de este proceso.

**Consideraciones Finales:**

urge una mayor inversión en políticas de formación y desarrollo profesional para facilitar la coordinación entre sectores, con miras a superar modelos pedagógicos y sanitarios obsoletos.

## INTRODUCTION

Learning difficulties are an educational problem that has emerged in the field of child and adolescent public health in recent decades, known as school complaints. Children who present this condition have been referred to specialized care, with the aim of resolving it under the medical eye, which has attracted health professionals’ and researchers’ attention^(1)^.

This difficulty is characterized by academic performance below expectations for students’ general conditions, as a result of multiple factors, isolated or in interaction, of endogenous and/or exogenous origin to children, which tends to disappear as its causes are remedied^(2)^. It differs from learning disorders or disorders, which are due to dysfunction of the central nervous system, of a functional nature, which involve a failure in the acquisition process or child development^(3)^.

The demand became more evident with elementary education universalization, revealing weaknesses in the teaching-learning process, especially in the age group between six and ten years old^(4)^. Educational statistics indicate that a large proportion of students have learning gaps from the first years of their school career. In Brazil, more than half of public school students reach the end of the third year of elementary school without knowing how to read, or reading poorly, with their school trajectory compromised. This is reflected in high failure rates, age-grade distortion, dropout and dropout rates^(2)^.

Thus, it must be considered that entering the education stage causes children to experience important changes in their development process that have repercussions on their relationships with themselves and others. In this regard, experiencing learning difficulties constitutes an obstacle to success in children’s school life, and the sooner it is identified, the greater the possibility of intervening and/or overcoming it, given that it can result in other problems, such as emotional (low self-esteem, lack of motivation) and family concern, in addition to repercussions in different spheres, such as personal, family, school and social^(5)^.

Currently, it is known that failure or success in school teaching and learning processes is less determined by individual issues than by institutional and political mechanisms^(6)^. Given these considerations, the research question emerged: what are the health and education professionals’ perceptions regarding learning difficulties in children enrolled in the public school system? It is important to know the care approaches carried out with children who experience difficulties in the teaching-learning process, considering different professional categories working in education and health networks, which justifies this study.

## OBJECTIVES

To understand health and education professionals’ perceptions regarding learning difficulties in children enrolled in the public school system.

## METHODS

### Ethical aspects

The recommendations established in Resolutions 466/2012 and 510/2016 and Circular Letter 2/2021 CONEP/SECNS/MoH were adopted, with approval by the Research Ethics Committee under Certificate of Presentation for Ethical Consideration (CAAE - *Certificado de Apresentação para Apreciação Ética*). To formalize acceptance of participation, the Informed Consent Form was signed, with guaranteed anonymity, with the names being replaced by the initials of the words “Health Professionals” and “Education Professionals” along with an Arabic numeral, such as HP1, HP2, EP1, EP2, and so on.

### Study design

This is qualitative research of the participant-action type based on Paulo Freire’s theoretical-methodological assumptions. Freire’s Research Itinerary^(7)^ was followed, which comprises three distinct and interconnected stages: 1) Thematic investigation: consists of the initial dialogue aimed at identifying the generating topics extracted from participants’ reality; 2) Coding and decoding: contextualization and problematization of generating topics occurs, expanding knowledge; 3) Critical unveiling: action-reflection process, becoming aware of the real situation, with a view to transforming the lived context^(8)^.

### Study setting

The study was carried out in four municipalities in the Greater Florianópolis Region, Santa Catarina, Brazil. The settings involved were the Health Care Network (RAS – *Rede de Atenção à Saúde*), in Primary Care, as it is considered the gateway to the Brazilian Health System (SUS – *Sistopic Único de Saúde*), and in Secondary Care, in two clinical services specialized in treating learning difficulties. In the Basic Education Network, included one institution from each municipality that assisted students in the early years of elementary school, in addition to one state and one federal institution, included because they are a reference in the educational area of that region.

### Data source

A total of 45 professionals participated, 27 from health and 18 from education. As inclusion criteria, professionals over 18 years old, working in care and/or management practice with children with learning difficulties, regardless of the type of employment relationship were included. Professionals with less than a year of employment, or who were away during the data collection period, were excluded. The selection process occurred for convenience, due to the proximity to the research problem, being indicated by those responsible for each institution. All invited professionals agreed to participate in the study, with no refusals.

### Data collection, organization and analysis

Data collection took place between November 2020 and April 2021. Due to the barriers imposed by the COVID-19 pandemic context, such as the need for social distancing and overload of activities in the health and education sectors, firstly, individual open-ended interviews were carried out with each professional, seeking to raise the generating topics for later discussion in a Virtual Culture Circle (VCC).

The interviews were previously scheduled by telephone, and carried out in person or virtually, at the choice of each professional, conducted by a nurse researcher with experience in this type of approach, lasting approximately one hour. The dialogue was triggered by guiding questions that addressed school learning difficulties and the relationship with social determinants of children and their families: I) What do you understand by school learning difficulties? II) What factors can interfere with the school learning process? III) How do you usually act when dealing with children with learning difficulties at school? IV) Is there any form of routing/segmentation for this situation? V) Do you notice or identify a relationship between health and learning difficulties? In what way? VI) What do you understand by the concept of social determinants of health?

Subsequently, a chart was created in a digital file with excerpts that reflected the generating topics extracted from professionals’ reality, constituting the thematic investigation. This stage represented the first moment of Paulo Freire’s itinerary.

The other stages took place in a Culture Circle, a term created by Paulo Freire, which involves the meeting between people or groups to reflect on emerging topics, with a view to building a deep perception of reality and developing collective intervention strategies^(9)^. For its development, the professionals who participated in the first stage were contacted remotely, however, due to incompatible schedules and/or vacations and licenses, only 21 professionals were able to be present, 10 from health and 11 from education. VCC occurred in a single session, lasting approximately two hours, mediated by a research nurse, with the support of two facilitators with experience in conducting this type of strategy.

To bring participants closer together, a brief presentation was held and, subsequently, the methodological proposal was contextualized, which, aiming to make it more playful and concrete, Paulo Freire’s Itinerary was analogized with the process of writing a book, which depends on different phases for its production, which are interdependent: thematic investigation corresponded to the survey of ideas about the history of be narrated; coding and decoding corresponded to content writing; and critical unveiling corresponded to the final work to be published.

In this way, the generating topics raised in the interviews were shared on the computer screen, in order to validate their meanings, promoting the action-reflection process. For the coding and decoding phase, the second stage of Research Itinerary, the mediator instigated the debate by talking about children’s learning difficulties, relating it to the topics raised in the interviews. From this, three generating topics were coded and decoded, which will be presented and discussed below.

Thus, participants experienced the last moment of Paulo Freire’s Itinerary, critical unveiling, in which it was possible to give new meaning to the three generating topics, relating the perception about the difficulty of school learning. The real possibilities of transforming the lived reality were discussed in VCC, which, through a process of action-reflection, socialized new perspectives for confronting learning difficulties in the context in which they worked.

The information was recorded through audio recording in on-site interviews using an application available on the smartphone as well as in virtual meetings, using the audiovisual resource available on Google Meet^®^. Subsequently, the information was transcribed, organized with the help of a Google Drive^®^ text editor and stored in digital folders.

Topic analysis occurred concomitantly with VCC development, during the completion of Research Itinerary stages, which foresees this continuous analytical process, which occurs with the interaction of all participants through reading, reflection and interpretation of emerging topics. The study followed the Consolidated criteria for REporting Qualitative research (COREQ) standards.

## RESULTS

Of the 45 participating professionals, 18 worked in the area of education and 27 in health, the majority of whom were female (41). The predominant age group was 41 to 50 years old, with 31 of them having a graduate degree at a specialization level, five with a master’s degree, two with a doctoral degree and seven with an undergraduate degree. Regarding the profession, 15 were pedagogues, seven were physicians, five were nurses, two were nursing technicians, five were social workers, three were speech therapists, three were dentists and three were psychologists.

Professionals chose three generating topics for discussion in VCC, namely: I) (In)visibility of learning difficulties; II) Educational sector strategies and resources; III) Search for resolution in the health sector.

In (In)visibility of learning difficulties, it was evident that professionals had different conceptions about the subject, being understood as a child who is unable to develop as expected for a certain age or school year, or who takes a little longer than others to learn, but who does not have a diagnosis, or who has difficulty reading or writing or in a specific discipline. In this regard, they discussed the need to differentiate learning difficulties and disorders, which are related to children’s neurobiological aspects, which are often considered synonymous by professionals.

*Difficulties are transitory problems. Children can start the year with a certain difficulty and, based on our observation, our assessment in the classroom, we notice, and we act more directly with it, investigating the possible cause. Then they transform, advance and improve their participation and learning. Disorders are something more specific, a very pronounced difficulty, which even has a neurological factor, a dysfunction in the central nervous system.* (EP10)

Some professionals highlighted the pedagogical/methodological issue, in which the education process is often not considered. They discussed that automatic promotion of students occurs in the first years of elementary school, understood as literacy cycle, with the perception of learning difficulties emerging from the third school year onwards.

*We have school as an automatic promotion literacy cycle. So, students don’t fail in the first or second year, they will fail in the third year, which is where the “boom” is happening. And children enter the first year at the age of six. In the third, they will be eight, which is where these difficulties will be most latent.* (HP24)

Furthermore, they identify difficulties among children in school based on observation of teachers in the classroom or other pedagogical team members. Occasionally, this perspective comes from the family or health professionals.

*Sometimes, for instance, the school that refers us asks for a specialized assessment or a diagnosis, or the family that realizes that they are having difficulty comes with this complaint, and sometimes we still end up making some perceptions during consultation.* (HP3)

They pointed out that students may demonstrate different signs to express difficulty, which may go unnoticed, confused with children’s neurobiological issues or even bad behavior:

*That child who can’t stay focused, pay attention in class, is always moving around, or is an apathetic child. Also, those who are the good children for some teachers, who do not identify with the group in the classroom, who are quiet, and there they remain quiet, then they are not remembered, these worry me a lot.* (EP16)

They highlighted that learning difficulties are a common situation in classrooms in public schools and a frequent complaint in health services, especially among students in the first years of elementary school. However, there is a lack of knowledge about the real proportion of this demand.

*In fact, we have no idea of the demand, which makes it very difficult to organize this situation. We have speeches, we talk about education, we talk about an Early Childhood Education Center, we talk about another service. Pediatricians come with this demand, but no one brings it to us like this* [...] *we would perhaps have to find a way to be able to understand this specific demand.* (HP19)

In the second generating topic, Strategies and resources in the educational sector, it was discussed that children need interventions aimed at developing their learning. Generally, teachers are primarily responsible for the initial approach. In this regard, they mentioned that they seek a change in learning strategy, teaching method in the classroom and/or providing individual care.

*In the first trimester, I do this survey, so who I can reach in my eyes, what they need to improve, I’m already helping. So, in my room, I’m working like this. I have a little friend who doesn’t even know “A”, this little friend will do this “A” activity. But for those who are literate, I will not give an “A” activity, I have another reading for that child in my class.* (EP1)

However, participants highlighted that this does not always happen, mainly due to overload of activities, excessive number of students in classes and due to lack of training to manage situations. If there is no progress in the face of the interventions carried out in the classroom, in a second moment, they revealed that the cases are discussed in pedagogical meetings. These are discussed with other teachers who work with children and/or directly with educational counselors, with a view to new interventions, often involving the family in this process.

*The teachers from the first to the fifth year ask me a lot, because sometimes there are three or four students in the classroom who are having difficulty. I try to provide support once a week, because I am alone at school to see what difficulties the child is having there, what is happening. I work with the child, I talk to the teacher to have greater interaction between us in relation to children, and when I call the family or guardians to talk, I see what is happening and ask to see if they can contribute* [...] *we can’t put all the responsibility on the family.* (EP2)

In schools where pedagogical support is available, they report that this more specific approach has favored the learning of many of these students. Moreover, they also mentioned the importance of community social projects, which often include activities of this nature after school.

*At the beginning of the year, I carry out a diagnostic assessment to find out where each child’s difficulties are, and then to be able to separate and plan the classes. Together with the teacher, we select students who need reinforcement, need guidance. It aims to help children who have learning difficulties avoid failing, and parents need to give permission. Then, students go to school, and twice a week they have literacy support.* (EP17)

They claimed that, for the most part, the public education network does not have a multidisciplinary team, whether internal or external to school, to support students and teachers. When available, joint education experts work to provide greater educational opportunities for children.

*We have a psychopedagogue, a pedagogue, a speech therapist and a psychologist. The demand is from the school, we assist children who are having some difficulty in the classroom during the after-school hours. Initially, we take the history, talk and then the investigation. We have a standard assessment of psychopedagogy and, depending on it, we carry out interventions, with playful activities that will help them develop. It’s a side job, not tutoring.* (EP6)

In other situations, this support is often limited to clinical services, such as assessment and direct support for students. However, they reported the need for collective interventions in the institution itself, which could collaborate.

*As head of the school, there would be more collective actions to promote learning, which I must say is difficult to carry out, because school professionals, in general, expect me to identify children who have changes to refer them to some service outside there. from school. This is difficult, I don’t see why I would only refer these students.* [...] *but routinely, I go to the classroom, observe some students that the teachers ask for, a closer look, I participate in class council meetings, grade meetings, in order to discuss the cases, and, most of the time, I do a lot of this referral role, I talk, I provide guidance to families.* (EP13)

They also mentioned that the presence of a multidisciplinary team is generally linked to special education, with a view to assisting students with some type of disability or specific disorder. However, they stated the importance of prioritizing inclusion approaches for all children who have learning difficulties at school.

*We started a project this year, which is a language workshop for some students who don’t have the diagnosis. Maybe they don’t have any of these formal ones, but they present a lot of difficulty, especially in reading, writing, solving problems in mathematics [...] but also trying to make actions more flexible with some children, who have various difficulties, some are multi-retained students, so we are concerned about also providing assistance to these children.* (*EP*12)

In the third generating topic, Search for resolution in the health sector, education professionals reported that, in cases where the approaches taken are insufficient and/or when children’s needs exceed the resources available in the sector, they refer families to seek care together to health services. They mentioned the importance of carrying out a neurological assessment in an attempt to find solutions for children’s non-learning.

*Sometimes, it’s really beyond the difficulty of what we can do in class. Then I talk to the counselor, we call the family, we write a report and ask them to go to the health unit to see the clinician to try a neurologist, then the neurologist will be able to make the other referrals that children really need.* (EP8)

The gateway to health services in the public network normally occurs through Primary Health Care, with demands regarding students’ learning difficulties being received during consultations or spontaneous demand services. In this context, when seeking to resolve the issue from the health sector, they infer that it is an individual factor of children that is influencing the process; however, they reported concern about this conduct, given that it can lead to a hasty or even erroneous diagnosis:

*I realize that there is a very big movement, an anguish of reaching a diagnosis due to learning difficulties, of having concrete data to be able to do something. And then after it arrives, what are we going to do with it? For me, the movement is reversed. We don’t need to label a developing child to be able to do something, because we can offer a more inclusive education model that meets families’ specificities and particularities in a cultural, social context, in short, with what they demand, without necessarily rushing a diagnosis. Because that child may be going through several situations that will influence them at that moment, but later they will develop their learning. And what we see most is a mistaken diagnosis of autism, attention deficit.* (EP11)

Furthermore, they discussed the growing trend towards medicalization of children who have learning difficulties, or do not behave in a way considered appropriate by schools. A fact that diverts and reduces the focus only to children’s biological dimension, and which can lead to other negative consequences for students.

*I work here a lot with school children and I see them going from year to year without the issue of literacy. I usually talk to families because then they go to the Health Unit wanting neuro or medicine because the child doesn’t learn. But the truth is that medicine does not replace processes, and what we see is that children go from one year to the next without the basic content, and then they arrive at the front understanding that they have dyslexia, a learning problem.* (HP17)

They warned that neurological issues, such as learning disorders, are existing problems, however they occur less frequently when compared to the number of referrals received. Thus, they highlighted the importance of assessments being carried out by multidisciplinary teams, whether in matrix meetings or through the School Health Program (SHP).

*It was a priority for discussions of situations* [of learning difficulties] *to take place in SHP meetings to first try to meet the demands in the territory, and when it went beyond that and we were unable to handle this service, then it would move on to another complexity.* [...] *a screening was carried out of what the school had already done in relation to education, and we as a health team carried out this clinical, ophthalmological, hearing screening, also family conflicts, presence of violence, negligence, lack of support, lack of encouragement.* (HP9)

Hence, they revealed that, by broadening their perspective on the situations that influence student learning, participants have found that, in addition to pedagogical and biological aspects, the social factor must be considered. It was evident that professionals perceive that an articulated and individualized intervention for each student with school learning difficulties is urgent, between the health and education sectors, in order to seek greater resolution to this demand.

## DISCUSSION

There is a certain vagueness in the concepts related to learning difficulties and disorders/disorders among professionals. This leads to the use of terms inappropriately, without often distinguishing their meaning. The lack of consensus on its understanding and definition is confirmed in the literature, as the main point of this disagreement lies in the fact that the population with learning difficulties is, in general, very heterogeneous^(10)^.

Furthermore, although the multifactorial nature of this phenomenon is recognized, responsibility for the situation generally falls on students, associated with a biological, emotional problem, or one arising from the social, structural and organizational aspects of their family, i.e., factors external to the school, as observed in studies^(4,11)^. This tendency minimizes the participation of social, political, economic and institutional relations in producing school complaints, disregarding the interference of teaching, mechanisms and school operations^(6)^.

It is noteworthy that pedagogical practices and educational policies appeared, less prominently, as corroborating this situation, which indicates a movement towards breaking this centrality in children and their families^(11)^. Continued progression policy weaknesses, which aims to prevent children from being retained in the first cycle of elementary school, favor students’ access and retention in school, without major distortions between age and grade. They are devoid of fundamental pedagogical measures for the teaching-learning process, such as different times and methodologies, reorganization of content, reduced number of students per classroom, service in small groups, outside class hours, for children who are late or have learning difficulties^(12)^.

Such an analysis can explain why complaints about learning difficulties manifest themselves in a greater proportion among children who attend the first years of elementary school, aged between seven and ten, with the highest percentage occurring in the third year, as highlighted by the participants of this study. Furthermore, the results showed a significant increase in the number of children referred to health care and specialized professionals, an increasingly frequent process nowadays, originating primarily from the public education network, with teachers being the main informants, as also seen in studies that address this topic^(6)^.

In view of these considerations, it was observed that, despite the theoretical-methodological advances proposed in new conceptions about school complaints, these are still not sufficiently present in these professionals’ practice^(6,11)^. The school has been uninteresting, and the teaching strategies used have not been adequate to students’ expectations, which ends up frustrating professionals involved in doing or thinking differently. With overcrowded classes, remuneration incompatible with the role, inadequate working conditions, there is an unfavorable professional reality for educators to carry out their work^(13)^.

The topic of learning difficulties seems distant from studies and practice carried out by educators, who are often unprepared to welcome students who face this situation, which demonstrates the relevance of the discussion in professional training. Furthermore, there is a lack of investment in important sectors, policies aimed at continuing education, inclusive school space processes and physical restructuring^(4)^.

However, although the perception that the teaching methodologies used by teachers in the classroom also produce learning difficulties and, consequently, academic failure is considered an advance, this view is questioned by authors who emphasize that moving from the dimension of blaming students and families to holding teachers accountable is also characterized as a reductionist view of the phenomenon. Educational policies and the Brazilian social project must be considered when understanding school failure^(14)^.

There is an urgent need to review the content-based pedagogical model, which is outdated and does not aim at students’ criticality. Structural issues that need attention were identified, as schools do not receive the necessary support from public authorities, such as the offering of after-school reinforcement programs to these students as well as from professionals to support teachers and students, such as psychologists, pedagogues, psychopedagogues and educational counselors^(11)^.

Despite the growing number of research in psychology and education that reaffirm the importance of identifying issues that go beyond individual and family dynamics of referred children, there remains a tendency by the professionals who assist them to treat school problems as exclusively biological or psychological in origin^(6)^. They attribute a central role to diagnosis as a determining element in the management of learning difficulties, placing their resolution in the health sector^(14)^.

As there are no real medical causes for school failure, there is an artificial construction of false relationships between illness and non-learning centered on individuals^(13)^. The naturalization of this process and continuous flow of production of demands for the health sector may signal a transfer of responsibilities from one area to another as a way of simplifying problems arising from a context certainly full of issues to be reevaluated and given new meanings, as previously pointed out^(6)^.

Attempts to deal with cases of school failure as if they were a pathology are examples of medicalization, i.e., a resource used to transform issues of eminently social and political origin into medical demands^(6)^. This represents a setback, as science has shown the ineffectiveness of the individual and medication approach to learning difficulties^(11)^.

The effectiveness of teaching and institutional dynamics are often not even questioned by health professionals, because they succumb to a superficial analysis of the situation of children and their families, especially those from the most impoverished classes, users of precarious schools^(6)^. As action is taken that values the medicalization process, only the person is treated and it is stated that the problem exists only in them, which generates a lack of responsibility for the various instances of power that produce and perpetuate such problems. However, understanding macrostructural factors (social, political) is fundamental to understanding the complexity of the multiple determinations of this phenomenon^(11)^.

On the other hand, the data showed that some professionals have incorporated a critical perspective, working to deconstruct stigmas and prejudices in relation to referred students. Thus, it was possible to detect the presence of a critical understanding of school complaint, implying assistance that aims to demystify and/or problematize the diagnosis, fundamentally in partnership with the school, which is essential for resolving learning difficulties^(15)^. Therefore, it is necessary that educators and health professionals have the necessary tools to understand children’s learning difficulties from an ethical and political perspective, in order to deconstruct the biomedical discourse that tends to be inserted into daily school life^(11)^.

It is proposed to value multiple learning and different school experiences as well as the importance of schools and teachers recognizing students’ reality, favoring the right to differences and singularities and avoiding labels and marginalization^(14)^. It is understood that the ideals of a democratic and secular school, i.e., of quality knowledge for all, constitute an objective yet to be achieved by our society^(4)^.

### Study limitations

The limitations of this study correspond to non-generalization of data, as it is a regional reality in the state of Santa Catarina, Brazil. It is worth mentioning that, due to health restrictions imposed by the COVID-19 pandemic, was impossible to carry out observation in the places of activity to monitor activities, especially in the educational field.

### Contributions to health and education

In light of these reflections, it is urgent to rethink the way of teaching, considering that each community has a different profile, characteristics and specific demands, requiring a certain adaptation from the teacher. The contributions to school health are highlighted, given the important role that professionals can play with individual and collective actions to promote students’ health and biopsychosocial well-being in order to favor children’s full development and academic success.

## FINAL CONSIDERATIONS

The study showed that learning difficulties are a situation influenced by several factors, but health and education professionals’ perspective tends to focus primarily on accountability for students and/or their families, and, eventually, for schools. Thus, a pathological characteristic has been attributed to this demand, seeking, together with health services, medicalizing strategies to solve it.

The perception that structural factors, such as social ones, also determine the phenomenon maintenance, in addition to the need to invest in educational policies for academic training and improvement in services emerged, but in an incipient form. In this regard, it is considered opportune to delve deeper into this topic in other research, involving students’ and their families’ understanding regarding school complaints, thus ensuring the expansion of knowledge in the complexity that the phenomenon demands.
